# Mental Health, Sense of Coherence, and Interpersonal Violence during the COVID-19 Pandemic Lockdown in Germany

**DOI:** 10.3390/jcm9113708

**Published:** 2020-11-18

**Authors:** Stefanie Jung, Jonas Kneer, Tillmann H. C. Krüger

**Affiliations:** 1 Department of Psychiatry, Social Psychiatry and Psychotherapy, Division of Clinical Psychology and Sexual Medicine, Hannover Medical School, Carl-Neuberg-Str. 1, 30625 Hannover, Germany; jung.stefanie@mh-hannover.de (S.J.); kneer.jonas@mh-hannover.de (J.K.); 2 Center for Systems Neuroscience, Bünteweg 2, 30559 Hannover, Germany

**Keywords:** COVID-19, coronavirus, lockdown, mental health, depression, interpersonal violence, sense of coherence

## Abstract

Preliminary data indicates that the Coronavirus SARS-CoV-2 disease (COVID-19) pandemic may have a substantial impact on mental health and well-being. We assessed mental health in response to the lockdown in Germany between 1 April 2020 and 15 April 2020 using a cross-sectional online survey (*n* = 3545) with a mixed-methods approach. We found increased levels of psychosocial distress (Patient Health Questionnaire (PHQ) stress module), anxiety, depressive symptoms (PHQ-4), irritability, and a decrease in overall well-being (WHO-Five Well-Being Index (WHO-5)), sense of coherence (Short Form of the Sense of Coherence Scale (SOC-L9)), sexual contentment, and sleep quality. The four-week-prevalence of interpersonal violence was yet at 5% and included verbal, physical, and sexual violence. Participants reported finding comfort in family, friends, conversation, exercise, and activity. Findings are also in line with research showing that women seem to have more trouble coping with the pandemic and lockdown measures. Our observations demonstrate that the COVID-19 pandemic and related measures lead to a mental health burden even in a highly developed Western country and should, therefore, be taken seriously. The findings for interpersonal violence are alarming. Thus, we should sharpen our focus on the matter and activate and enhance supporting systems to help protect those affected.

## 1. Introduction

The novel, highly contagious corona virus SARS-CoV-2 is currently spreading all over the world. First cases of Coronavirus SARS-CoV-2 disease (COVID-19) were reported in Wuhan, China, in early December 2019. Symptoms mainly include respiratory distress, fever, coughing, and fatigue. As of 13 November 2020, 53,126,651 cases of COVID-19 and 1,312,170 COVID-19 related deaths have been confirmed worldwide (for Germany: 771,976 cases of COVID-19 and 12,270 COVID-19-related deaths) (John Hopkins University) [1]. The diseases’ course has, thus, proven to be potentially fatal. In order to flatten the curve, measures like social distancing, wearing of a protective mask, enhanced hygiene concepts, and temporary lockdown have been taken in most countries worldwide. The current focus on COVID-19 infections, however, might distract attention from mental health issues related to the outbreak and the measures taken in order to prevent further spreading [2]. In fact, besides its impact on physical health for those infected, the pandemic and the measures taken seem to have a substantial impact on mental health and well-being. Preliminary data from China indicated increased levels of psychological distress, anxiety, depressive symptoms, and insomnia [3,4]. Another Chinese survey found that more than half of the general population rated the outbreak’s psychological impact as moderate or severe [5].

Studies related to mental health during the COVID-19 pandemic are still scarce, but more results indicate that anxiety and depression increase with an overall decrease in psychological well-being [6]. Increasing feelings of isolation, fear, worry, and sadness may cause depression and abuse of alcohol, drugs, and prescription medication and may also lead to violence toward the self or others [7]. Some authors even go so far as to describe the current situation as a public mental health crisis [8], the next mental health pandemic [9], or a mental health emergency [10]. Factors associated with a current decrease in mental health include female gender, lower socioeconomic status, lower education, and poor sleep quality [6]. Pandemic-related symptoms of depression and post-traumatic stress disorder (PTSD) have also been found to be linked to female gender and lower socioeconomic status [11].

In March 2020, the German government agreed upon a substantial catalogue of lockdown measures including contact bans that came into effect on 22 March 2020. Such measures were unprecedented for the majority of people and may affect their lives tremendously. As for now, few studies from Europe have been published [6] and yet it seems of vital importance to surveil the psychosocial consequences of the current pandemic. Here, we present data that was taken during the height of lockdown measures in Germany from 1 April 2020 to 15 April 2020.

For one, we assume an increase in depression, anxiety, impaired sleep, and domestic violence as well as changes in alcohol and food consumption. Measures taken against the spreading of the coronavirus included a strict social distancing protocol that also meant a nationwide lockdown with people being advised to stay at home and to only leave the house for essential activities such as work, shopping for essential goods, and care of pets. These measures lead to an involuntary decrease in social contact. Lack of contact with other humans can cause feelings of loneliness, which may lead to depression [12], negative self-esteem, anxiety, feeling unsafe [13], and impaired sleep [14,15]. Moreover, perceived loneliness impairs the capacity to self-regulate [16], which could lead to dysfunctional behavioral changes such as an increase in alcohol or food consumption [17,18]. In addition, when combined with confined domestic circumstances, a decrease in self-regulation could lead to an increase in domestic violence. Second, we assume that the sense of coherence decreased, leaving the German population vulnerable to stress. Sense of coherence is a theoretical concept that offers a framework for overall coping in life. It combines three key aspects: comprehensibility, manageability, and meaningfulness [19]. Whether we understand the things happening to us, whether we believe that we have the necessary resources and skills to manage, and whether things in life are worthwhile and have a purpose, greatly defines how we cope with and how we perceive stressful events. We believe that the pandemic and the measures taken against it constitute such a stressful event. At the beginning of the pandemic, there was little understanding concerning why and how the virus spread or which measures were useful. The uncertainty and the lockdown may have increased feelings of powerlessness and, thus, reduced feelings of manageability.

The current survey was developed in order to systematically assess mental health in response to the pandemic and the measures taken in order to contain it. We sought out to replicate and expand findings from China for the German population and to explore perceived risks and remedies in order to derive much-needed implications for politics and healthcare.

## 2. Experimental Section

The development of the current study started as the COVID-19 pandemic gathered speed and an implementation of lockdown measures in Germany was starting to become conceivable. Since the population was advised to stay at home, we agreed upon conducting an online survey. We put together a test battery including quantitative as well as qualitative measurements. Apart from demographics, quantitative instruments included the Patient Health Questionnaire-4 (PHQ-4), the Patient Health Questionnaire stress module (PHQ stress module), the WHO-5 Well-being Index (WHO-5), and the Sense of Coherence Scale–short form Leipzig (SOC-L9). Moreover, using comparative questions on 3-point and 5-point Likert scales, we asked participants to indicate changes to pre-lockdown times (workplace changes, feelings of aggression, sleep quality, quality and quantity of nutritional intake, quality and quantity of sexual activity, availability of time, and experience of violence). We constructed the comparative questions using a multi-step procedure. After reviewing current research from China that already pointed toward the psychosocial areas that might be affected by the pandemic and the measures taken, we brought together the first set of items. Initial items were subsequently revised by experts and members of our department until we reached agreement upon the additional items concerning changes to pre-lockdown times. Additionally, we included a multiple selection question concerning how participants spend their time and two open-ended questions concerning what helped participants during this time and which opportunities they expected to stem from the COVID-19 pandemic and the measures taken against it.

### 2.1. Patient Health Questionnaire-4 (PHQ-4)

The PHQ-4 briefly measures anxiety and depression. It consists of the first two items of the Generalized Anxiety Disorder-7 scale (GAD-7) and the Patient Health Questionnaire-8 (PHQ-8) and shows good reliability [20].

### 2.2. Patient Health Questionnaire Stress Module (PHQ Stress Module)

The PHQ stress module is a 15-item questionnaire measuring psychosocial risk factors that contribute to the development of psychiatric disease. It consists of the PHQ items 12a–12j and shows good reliability [21].

### 2.3. WHO-5 Well-Being Index (WHO-5)

The WHO-5 is a five-item scale measuring current mental well-being while referencing the previous two weeks. It shows high clinometric validity [22].

### 2.4. Sense of Coherence Scale—Short Form Leipzig (SOC-L9)

The SOC-L9 is a nine-item short form scale derived from the original 29-item questionnaire. It measures sense of coherence, which is a construct referring to a person’s attitude or confidence that intrapsychic and environmental events are predictable and manageable. Sense of coherence is believed to be a protective factor for mental health. The scale shows good reliability and validity [23].

Access to the online survey was spread and made available through social media (Instagram, Facebook), mailing lists, the Hannover Medical Schools’ website, and TV as well as radio appearances by T.H.C. Krüger. Participants from 18 years up were invited to participate. Deliberately, there were no further inclusion and exclusion criteria as we sought out to reach as many citizens as possible.

### 2.5. Statistical Analysis

Data was analyzed using SPSS Statistics 26 (IBM^®^ Corporation, Amonk, NY, USA) and tested for normal distribution and non-violence of assumptions, which were applicable prior to further analysis. We mainly report means and standard deviations, group comparisons (using *t*-tests and Mann-Whitney-U-tests with Bonferroni-Holm adjustment), and frequencies (in percent).

### 2.6. Analysis of Qualitative Data

Our qualitative data analysis was guided by qualitative content analysis [24]. After sifting through raw data in order to get an overview, filler words like and, the, in… were excluded from further analysis. After data cleansing, we applied the summary method that aims at reducing the material in a way in which essential content is retained. Using abstraction, we build a corpus that represents the raw material and clustered keywords into contextual theme blocks. Our coding was inductive. Thus, categories were derived from raw data. Since the sample size for men is rather small and the gender differences we found showed only small effect sizes, we will report qualitative data for women and men as one sample.

The survey was approved by the local ethics committee at Hannover Medical School, Germany (Nr. 9002_BO_K_2020). Data was collected during the height of lockdown measures in Germany from 1 April 2020 to 15 April 2020. Participants were informed about the survey content and consented by starting the questionnaire.

## 3. Results

Demographics. A total of 3545 volunteers took part in this cross-sectional survey. Mean age was M = 40.36 years, standard deviation (SD) = 11.70, *n* = 2946 (83.1%) female, *n* = 539 (15.2%) male, *n* = 60 (1.7%) diverse or missing), mean educational years 15.87 (SD = 4.19), 30.6% held a university degree, 9.9% were unemployed, and 23.9% reported living alone. Acute or chronic disease was reported by 36.7% (physical) and 24.7% (mental) of subjects. Mean duration for completion of the survey was at M = 1134.53 seconds (18.9 min) (SD = 575.35 seconds, 9.6 min). Due to the imbalanced gender distribution, we will report further results separately for women and men.

**Figure 1:**
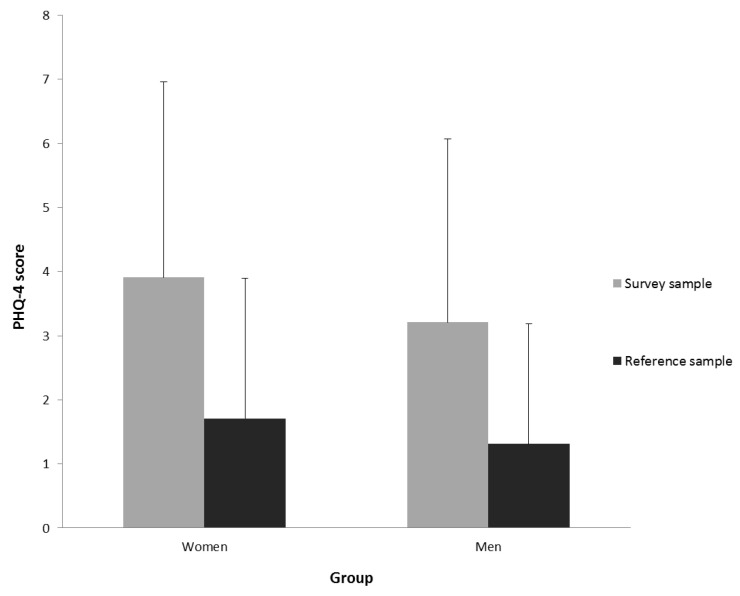
Anxiety and Depression (Patient Health Questionnaire (PHQ-4)): Means and standard deviation of PHQ-4 score for depression and anxiety for women and men in the reference and survey sample.

**Figure 2:**
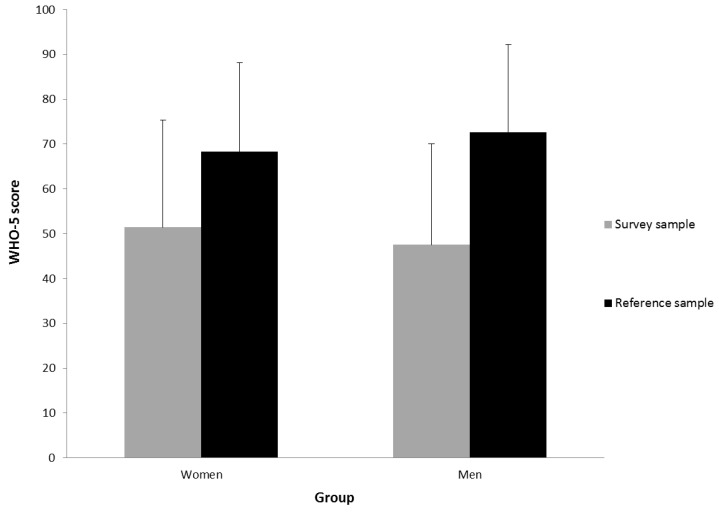
Well-being (WHO-Five Well-Being Index (WHO-5)): Means and standard deviation of WHO-5 well-being score for women and men in reference and survey sample.

Depression, Anxiety, and Distress. Depression and anxiety as assessed by PHQ-4 was at M = 3.91 (SD = 3.05) for women and at M = 3.21 (SD = 2.86) for men. Reference samples show mean scores of M = 1.71 (SD = 2.19) for women and M = 1.31 (SD = 1.88) for men [25]. Thus, PHQ-4 scores were significantly higher in our sample for both genders ((*t*(4254) = −23.66, *p* < 0.001) for women and (*t*(1700) = −16.28, *p* < 0.001) for men with Bonferroni-Holm-adjustment). Psychosocial distress as measured with the PHQ stress module was at M = 6.40 (SD = 3.88) for women and at M = 6.19 (SD = 4.00) for men, implying mild psychosocial distress (range 5–9) for both genders. The mean well-being score (WHO-5) was at M = 51.44 (SD = 23.88) for women and at M = 47.52 (SD = 22.52) for men (range 0–100). Healthy individuals usually score at M = 75.00 and subjects with major depression usually score at M = 37.50 (WHO, 1998) [26]. Brähler et al. [27] reported the following values for the psychometric validation and standardization of the WHO-5 German version: M = 72.6, SD = 4.90 for men and M = 68.28, SD = 4.98 for women. See Figure 1 and Figure 2. Bonferroni-Holm-adjusted calculation of gender differences revealed higher scores for depression and anxiety (*t*(3459) = 4.93, *p* < 0.001), but also a higher well-being score ((*t*(3451) = 3.52, *p* < 0.001)) in women. Effect sizes reported as Cohen’s d, however, demonstrate small effects with *d* = 0.24 for depression and anxiety and *d* = 0.17 for well-being.

Sense of Coherence and Coping. Sense of coherence as measured with SOC-L9 was at M = 41.77 (SD = 10.07) for women and at M = 44.11 (SD = 9.71) for men. Reference samples show mean scores of M = 46.70 (SD = 9.00) for women and M = 48.50 (SD = 8.80) for men [28]. Thus, SOC-L9 scores were significantly lower in our sample for both genders (*t*(3756) = 14.17, *p* < 0.001), for women (*t*(1372) = 8.56, *p* < 0.001), and for men with Bonferroni-Holm-adjustment). The majority of subjects (58.1% women, 70.3% men) indicated very good or fairly subjective coping with the pandemic and the corresponding measures, while 28.7% women and 18.2% men indicated poor or very poor subjective coping. Bonferroni-Holm-adjusted calculation of gender differences revealed poorer subjective coping (U = 678156, *p* < 0.001) and lower sense of coherence (*t*(3125) = −4.75, *p* < 0.001) in women. Effect sizes reported as Cohen’s d and Pearson’s r, however, demonstrate small effects with *d* = 0.24 for sense of coherence and *r* = 0.1 for coping.

Sleeping, Eating, and Sexual Activity. Using comparative questions, 46.5% of all women and 39.5% of all men indicated worsened sleep compared to pre-pandemic times. Bonferroni-Holm-adjusted calculation of gender differences revealed poorer sleep quality in women (U = 740746, *p* < 0.05), even though the effect size shows a small effect (*r* = 0.05). Of all women, 29.2% reported eating less healthy, compared to 18.9% reporting eating healthier. Moreover, 38.1% reported eating more, compared to 16.2% reporting eating less. Of all men, 23.6% reported eating less healthy, compared to 18% reporting eating healthier. Moreover, 26.3% reported eating more, compared to 14.5% reporting eating less. Regarding sexual activity, 20.5% of all women reported having less sexual intercourse, compared to 7.5% reporting having more sexual intercourse. Of all men, 20.4% reported having less sexual intercourse, compared to 7.4% reporting having more sexual intercourse. Furthermore, 21.6% of all women reported decreased sexual contentment, compared to 4.6% reporting increased sexual contentment. Of all men, 29.5% reported decreased sexual contentment, compared to 4.1% reporting increased sexual contentment.

### 3.1. Anger and Violence

#### 3.1.1. Women

A total of 22.8% of all women reported being slightly more easily angry/aggressive, compared to 6.7% reporting feeling slightly less easily angry/aggressive. Furthermore, 7.5% of all women reported experiencing way more anger and aggression, compared to 6.3% experiencing way less. Of those women, who experienced way more anger and aggression, 66.2% directed their anger and aggression at others, while 33.8% directed it at themselves.

#### 3.1.2. Men

Additionally, 20.2% of all men reported being slightly more easily angry/aggressive, compared to 7.6% reporting feeling slightly less easily angry/aggressive. In addition, 2.6% of all men reported experiencing way more anger and aggression, compared to 4.8% experiencing way less. Of those men, who experienced way more anger and aggression, 71.9% directed their anger and aggression at others, while 28.1% directed it at themselves.

Most importantly, 5% of all participants (5.1% of all women and 4.1% of all men) reported experiencing interpersonal violence (IPV) on a verbal (98% of all women and 100% of all men who experienced IPV), physical (38.4% of all women and 63.6% of all men who experienced IPV), or a sexual (26.5% of all women and 50% of all men who experienced IPV) level. In case of verbal violence, 76.8% of all women and 78.2% of all men reported experiencing more verbal violence lately. Regarding physical violence, 14.5% of all women and 21.4% of all men reported experiencing increased levels and, in case of sexual violence, 2.6% of all women but none of the men reported experiencing increased sexual violence lately. Of note, Bonferroni-Holm-adjusted calculation of gender differences revealed more experience of physical violence in men (U = 1206, *p* < 0.05), even though the effect size demonstrates a small effect with *r* = 0.18.

Pastime. While women reported to mostly spend their time doing household chores (65.2%), cooking (54%), and watching movies/TV (50.1%), men reported to mostly spend their time watching movies/TV (51.9%), working (47.3%), and doing household chores (46.6%) (also see Table 1).

Pre-existing mental and physical health conditions. The results show that 24.7% of participants reported pre-existing mental health issues. In order to analyze the impact on our outcome measures, we compared participants with and without mental disease. Bonferroni-Holm-adjusted calculation of differences revealed more anxiety and depression (PHQ-4) in participants with pre-existing mental conditions (U = 548645, *p* < 0.001) with a small to medium effect with *r* = 0.39. Moreover, Bonferroni-Holm-adjusted calculation of differences revealed lower well-being (WHO-5) in participants with pre-existing mental conditions (U = 582613, *p* < 0.001) with a small to medium effect with *r* = 0.37. Furthermore, Bonferroni-Holm-adjusted calculation of differences revealed a lower sense of coherence (SOC-L9) in participants with pre-existing mental conditions (U = 378028, *p* < 0.001) with a small to medium effect with *r* = 0.44. Additionally, Bonferroni-Holm-adjusted calculation of differences revealed worse coping in participants with pre-existing mental conditions (U = 830145, *p* < 0.001), even though the effect size demonstrates a small effect with *r* = 0.23. Lastly, Bonferroni-Holm-adjusted calculation of differences revealed more stress (PHQ stress module) in participants with pre-existing mental conditions (U = 933171, *p* < 0.05), even though the effect size demonstrates a very small effect with *r* = 0.04 (also see Table 2, Table 3 and Table 4). Pre-existing physical health conditions were reported by 36.7% of participants. Although we found statistically significant differences for outcome measures between participants with and without pre-existing physical health conditions, the effect sizes indicate negligible effects (see Tables S1–S3).

### 3.2. Qualitative Data

What does help you during the COVID-19 pandemic? With 35.3%, participants mainly reported finding comfort in their families (including partner and children). Talking to others (18.3%), friends (17.3%), exercise (15.5%), and staying active/occupied and distracted (14.9%) were also perceived as helpful (also see Table 5).

Opportunities stemming from the COVID-19 pandemic. The biggest perceived opportunity stemming from the current pandemic seems to be appreciation/thankfulness (19.6%). Moreover, participants believed that environmental and climate protection (16.1%) as well as reevaluation and rethinking current values (15.1%) and solidarity/willingness to help (11.4%) might be positive outcomes. Beyond, participants named health care system (9.2%), society/community (7.8%), and together (7.4%) as opportunities stemming from the ongoing pandemic (see Table 6).

## 4. Discussion

This is one of the first and largest surveys on mental health during the current COVID-19 pandemic in a European society. Although the cohort reflects a relatively well educated and financially secure sample, we found evidence for a substantial mental burden with increased levels of psychosocial distress, irritability (anger/aggression), anxiety, and depressive symptoms. Moreover, participants reported overall lower well-being, lower sense of coherence, decreased sexual contentment, less healthy diet, and almost half of the sample indicated worsened sleep. Our results further indicate that participants with pre-existing mental conditions show more depression and anxiety, less well-being, less sense of coherence, and worse coping skills in terms of the pandemic and the measures taken. Most importantly and also most concerning is the finding of a one-month prevalence of 5% IPV, which is already close to one-year prevalence rates [29] and for which there were indices that this has currently increased. Both women and men experienced more anger and aggression. Both predominantly directed their anger at others rather than themselves.

While we do not present an analysis of risk factors, in line with current research [6], we found that women showed higher levels of anxiety and depression and worse coping, even though small effect sizes restrict these findings. One explanation, however, might be that women seem to lapse into traditional roles. While women report to predominantly spend their time with household chores and cooking, men mainly reported watching movies/TV and going to work. It seems conceivable that an imbalanced distribution of child care and household responsibilities led to fear of or actual work place changes for women, which might, in turn, have further increased women’s burden. 

Besides the negative impact the current COVID-19 pandemic seems to have on our world population, participants also reported opportunities and pointed out what seemed helpful and beneficial. Almost one-fifth of the sample noted that the current pandemic offers the opportunity to be thankful for and appreciate what we have with 15% assuming that a process of reevaluation might take place. As many as 16% believe that the situation might lead to a sharpened view for environmental and climate issues. More than one-third found comfort in their families, children, and life partners. Almost one-fifth experienced talking to others and friends as beneficial. Exercise and staying active also seemed to help people coping with the COVID-19 pandemic.

The current study offers several strengths and limitations. While some authors argue that sample sizes between *n* = 1000 and *n* = 3000 are usually sufficient enough to make assertions for the general population [30], despite the large sample size, norm deviations for various demographics lead to limitations concerning representativeness. First, in our data set, we found a large gender imbalance. The German population consists of 50.66% women and 49.34% men [31]. Yet, in our study, we found 83.1% females and 15.2% males. However, the phenomenon of gender imbalance in online surveys with women representing the majority is established, as women are more likely to participate in online surveys [32]. Moreover, gender imbalance has been observed in other recent COVID-19 online surveys. Di Renzo et al. [33] reported 80% female respondents and Hsing-Ying Ho et al. [34] reported 66% female respondents. Further potential explanations might be the following: (1) women are more likely to use social media [35] (2) with women staying more at home during the lockdown than men, they had the occasion to participate in surveys, (3) the title of the survey included the term “mental health,” which may have spoken more to women than to men, and (4) women are impacted by affective disorders twice as much as men, so they may have had a higher incentive to participate. Second, our sample was well educated. While 17.6% of the German population hold a university degree [36], in our sample we found 30.6% with a Bachelor’s degree, a Master’s degree or diploma. The unemployment rate in our sample was higher than in the German population (9.9% in our sample and 6.2% in the German population [37]). Mean age (40.36 years in our sample and 44.5 years in the German population [38]) and household status (23.9% reported living alone in our sample vs. 21.13% single households in Germany [39]), however, did not seem to differ widely. Taken together, the demographic deviations from the norm restrict representativeness. Moreover, we did not investigate depression and anxiety rates before the pandemic. Thus, we can only draw a comparison between current pandemic values and reference values. We did, however, check for pre-existing mental and physical illness. Concerning pre-existing mental conditions, the occurrence corresponds to point prevalence in Germany [40]. Thus, a resulting bias cannot be expected. Further on, we did not explore the extent to which women were involved in child care during the lockdown, which included closing of day care and schools. Beyond that, we did not control for social desirability effects. These aspects should be taken into account for future studies. Yet, using a mixed-methods approach, we present comprehensive European data that gives a valuable insight into potential challenges and protective factors for mental health during the height of lockdown measures in Germany. 

## 5. Conclusions

Although there is reason to expect that mental health will increase with the successful containment of the COVID-19 pandemic [41], the spreading course of the coronavirus seems to be coming and going in waves. The populations’ mental health can, thus, be assumed to be equally dynamic. The latest numbers from the U.S., for example, demonstrate that, related to the pandemic, 40% of respondents showed signs of anxiety, depression, or increased use of substances with 25% even reporting symptoms of trauma-related and stressor-related disorders [42]. By now, COVID-19 case numbers worldwide have gone up again and measures are being intensified globally. Therefore, it is of vital importance to continuously monitor the mental health of the general public during this pandemic and its aftermath to identify associated protective factors and to carefully screen for IPV and its risk factors such as stress, sleep problems, and anger [43].

## Figures and Tables

**Table 1:** Pastime.

Women		Men	
Activity	%	Activity	%
1. Household Chores	65.2	1. Watching Movies/TV	51.9
2. Cooking	54.0	2. Work	47.3
3. Watching Movies/TV	50.1	3. Household Chores	46.6
4. Taking A Walk	45.0	4. Spending Time with Family/Partner	
5. Spending Time with Family/Partner	43.8	5. Reading/Watching News	41.9
6. Work	42.3	6. Playing on the Computer/Console/Smartphone	38.0
7. Cleaning	39.9	7. Taking a Walk	36.7
8. Reading/Watching News	37.6	8. Distraction Using Media	35.8
9. Write Text Messages/Chat with Friends/Family	37.1	9. Cooking	35.3
10. Chat with Friends/Family Via Telephone/Video	34.9	10. Listening to Music	28.8

*Notes*. *n* = 3545, *n* = 2946 female, *n* = 539 male.

**Table 2:** Mann-Whitney-U-tests for group differences between participants with (WMHC) and without (WOMHC) pre-existing mental health conditions.

Variable	*z*-Value	*p*-Value	*r*-Value	Mean Rank _WMHC_^a^	Mean Rank _WOMHC_^b^
PHQ-4	−23.22	<0.001	0.39	2438.65	1526.21
WHO-5	−21.61	<0.001	0.37	2389.74	1536.61
SOC-L9	−24.88	<0.001	0.44	875.40	1813.63
Coping	−13.46	<0.001	0.23	2140.43	1638.00
PHQ stress	−2.57	0.035	0.04	1703.36	1605.70

*Notes*. *^a^* = with mental health condition, *n* = 877, *^b^* = without mental health condition, *n* = 2651. PHQ-4 = Patient Health Questionnaire-4, WHO-5 = WHO-5 Well-being Index, SOC-L9 = Sense of Coherence Scale–short form Leipzig, PHQ stress module = Patient Health Questionnaire stress module.

**Table 3:** Means and standard deviations for PHQ-4, WHO-5, SOC-L9, and PHQ stress module for participants with (WMHC) and without (WOMHC) pre-existing mental health conditions.

Variable	M_WMHC_^a^	SD_WMHC_^a^	M_WOMHC_^b^	SD_WOMHC_^b^
PHQ-4	6.02	3.34	3.06	2.53
PHQ Stress	6.67	3.95	6.26	3.87
WHO-5	16.50	5.43	11.43	5.55
SOC-L9	33.98	9.69	44.79	8.67

*Notes*. *^a^* = with mental health condition, *n* = 877 *^b^* = without mental health condition, *n* = 2651. PHQ-4 = Patient Health Questionnaire-4, PHQ stress module = Patient Health Questionnaire stress module, WHO-5 = WHO-5 Well-being Index, SOC-L9 = Sense of Coherence Scale–short form Leipzig.

**Table 4:** Answers for item Coping (in percentage) for participants with (WMHC) and without (WOMHC) pre-existing mental health conditions.

How Well Are You Coping?	Percentage %_WMHC_^a^	Percentage %_WOMHC_^b^
Very good	8.1	16.2
Good	34.3	49.8
Neither nor	13.9	12.6
Not very good	33.8	18.5
Not good at all	9.9	2.9

*Notes*. *^a^* = with mental health condition, *n* = 877 *^b^* = without mental health condition, *n* = 2651.

**Table 5:** What does help you during the COVID-19 * pandemic?

Theme	*n*	%
Family/Partner/Children	1036	35.3
Conversation/Communication	538	18.3
Friends	507	17.3
Exercise	456	15.5
Distraction/Activity/Occupation	437	14.9
Contact	179	6.1
Having More Time	167	5.7
Garden/Nature	143	4.9
Pets	133	4.5
Fine Weather	109	3.7
Nothing	98	3.3

*Notes*. We will report data up to 3%. *n* = 3545 with *n* = 606 not stated, percentage related to remaining *n* = 2939. * Coronavirus SARS-CoV-2 disease.

**Table 6:** Opportunities stemming from the COVID-19 * pandemic.

Theme	*n*	%
Appreciation/Thankfulness	553	19.6
Environmental Protection/Climate Protection	453	16.1
Reevaluation/Rethinking/Consciousness	425	15.1
Solidarity/Willingness to help	322	11.4
Healthcare System/Care-Giver	260	9.2
Society/Community	219	7.8
Together	209	7.4
Slow Movement	163	5.8
Home Office	137	4.9
Family	114	4.0
Digitalization	109	3.9

*Notes*. We will report data up to 3%. *n* = 3545 with *n* = 723 not stated, percentage related to remaining *n* = 2822. * Coronavirus SARS-CoV-2 disease.

## Supplementary Materials

The following are available online at https://www.mdpi.com/2077-0383/9/11/3708/s1, Table S1: Mann-Whitney-U-tests for group differences between participants with and without pre-existing physical health conditions, Table S2: Means and standard deviations for PHQ-4, WHO-5, SOC-L9 and PHQ stress module for participants with and without pre-existing physical health conditions., Table S3: Answers for item “coping” (percentage) for participants with and without pre-existing physical health conditions.
