# Supplementary material for: Mental Health, Sense of Coherence, and Interpersonal Violence during the COVID-19 Pandemic Lockdown in Germany
Source: J Clin Med. 2020 Nov 18;9(11):3708. doi: 10.3390/jcm9113708 (PMC7699150; doi:10.3390/jcm9113708)
Supplement: Supplementary file 1 [file jcm-09-03708-s001.pdf]

Journal of
Clinical Medicine

Supplementary Materials

Table S1. Mann-Whitney-U-tests for group differences between participants with and without pre-existing physical health conditions.

Variable	z-Value	p-Value	r-Value	Mean Rank $_{WPHC}$ ^{a}	Mean Rank $_{WOPHC}$ ^{b}
PHQ-4	−6.69	0.000	0.11	1903.16	1667.42
WHO-5	−7.58	0.000	0.13	1919.06	1651.04
SOC-L9	−5.89	0.000	0.10	1457.43	1656.52
Coping	−2.36	0.018	0.04	1813.88	1734.92
PHQ stress	−2.75	0.006	0.05	1690.17	1596.60

Notes. ^{a} = with physical health condition, n = 1300 ^{b} = without physical health condition, n = 2230.

Table S2. Means and standard deviations for PHQ-4, WHO-5, SOC-L9 and PHQ stress module for participants with and without pre-existing physical health conditions.

Variable	M_{WPHC}^{a}	SD_{WPHC}^{a}	M_{WOPHC}^{b}	SD_{WOPHC}^{b}
PHQ-4	4.26	3.20	3.52	2.90
PHQ Stress	6.61	3.92	6.23	3.87
WHO-5	13.70	5.97	12.10	5.84
SOC-L9	40.68	10.31	42.95	9.83

Notes. ^{a} = with physical health condition, n = 1300 ^{b} = without physical health condition, n = 2230.

Table S3. Answers for item "coping" (percentage) for participants with and without pre-existing physical health conditions.

How Well Are You Coping?	Percentage $\%_{WPHC}$ ^{a}	Percentage $\%_{WOPHC}$ ^{b}
Very good	14.1	14.3
Good	43.5	47.3
Neither nor	12.8	13.0
Not very good	23.8	21.4
Not good at all	5.8	3.9

Notes. ^{a} = with physical health condition, n = 1300 ^{b} = without physical health condition, n = 2230.

© 2020 by the authors. Licensee MDPI, Basel, Switzerland. This article is an open access article distributed under the terms and conditions of the Creative Commons Attribution (CC BY) license (http://creativecommons.org/licenses/by/4.0/).
